# Supplementary material for: Construction of Engineered Muscle Tissue Consisting of Myotube Bundles in a Collagen Gel Matrix
Source: Gels. 2023 Feb 8;9(2):141. doi: 10.3390/gels9020141 (PMC9956229; doi:10.3390/gels9020141)
Supplement: Supplementary file 1 [file gels-09-00141-s001.zip › gels-2192560-supplementary.pdf]

Supporting Information

Construction of the engineered muscle tissues consisted of myotubes bundled with the collagen gel matrix

[1,*]Kazuya Furusawa, [1]Yuki Kawahana, [1]Ryoya Miyashita

[1]Department of Applied Chemistry and Food Science, Faculty of Environmental and Information Science, Fukui University of Technology

Correspondence information: *To whom correspondence should be addressed

E-mail:kfurusawa@fukui-ut.ac.jp. Tel.: +81-776-29-2465

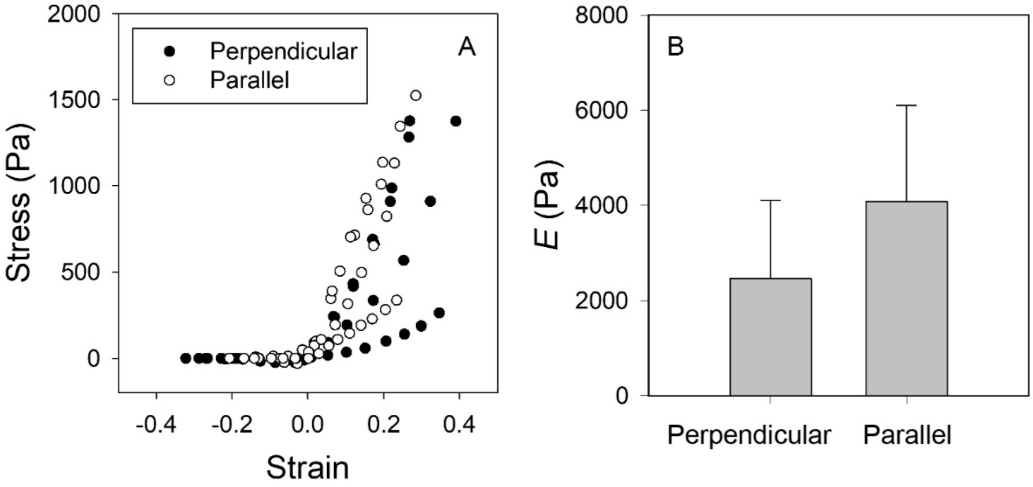

Figure S1. A: Stress – Strain curves of a chicken thigh (CT) in the perpendicular and the parallel directions. All measured results are summarized in the figure (*n* = 4). B: Comparison of elastic moduli of CT. The error bar indicates standard deviation (*n* = 4).

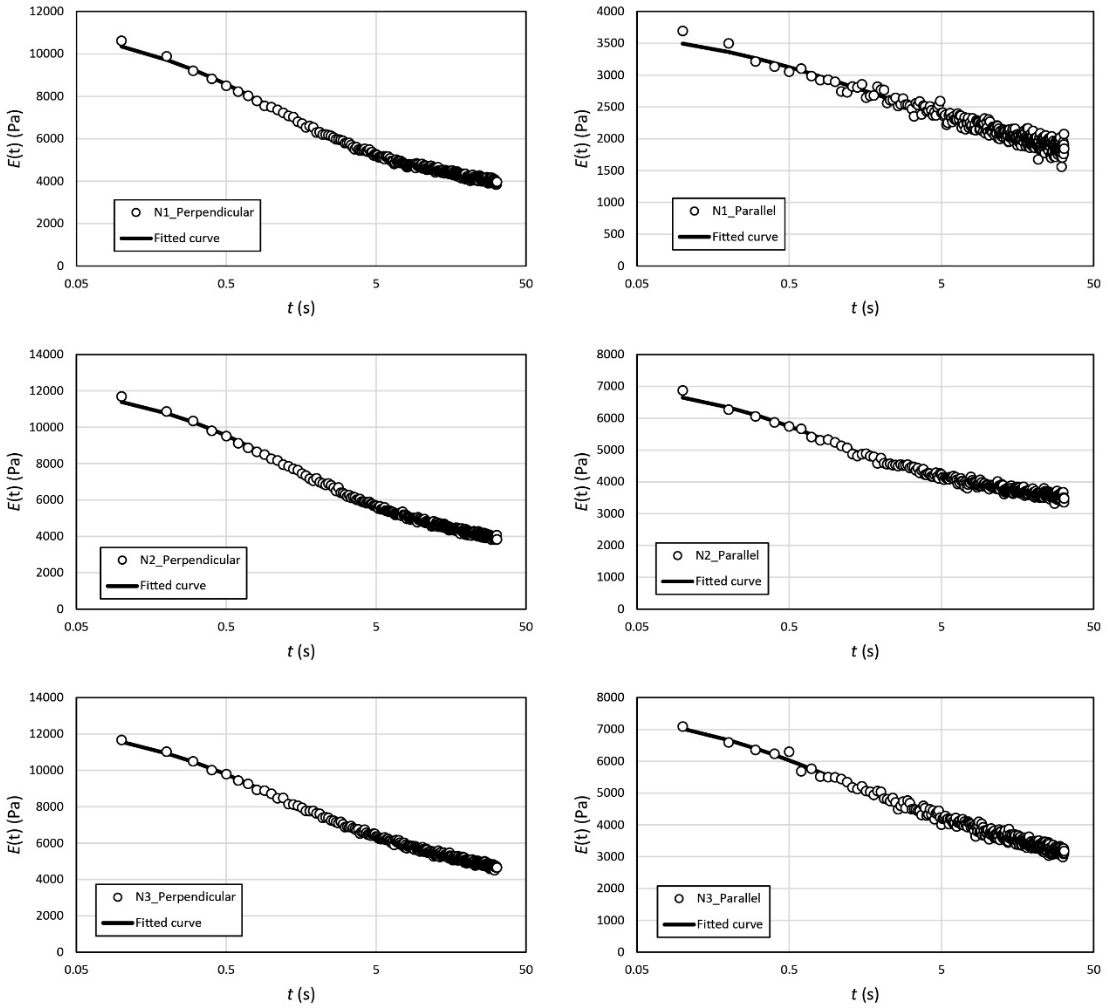

Figure S2. Curve fitting the relaxation moduli of EMT in the perpendicular and the parallel directions with the equation 3.

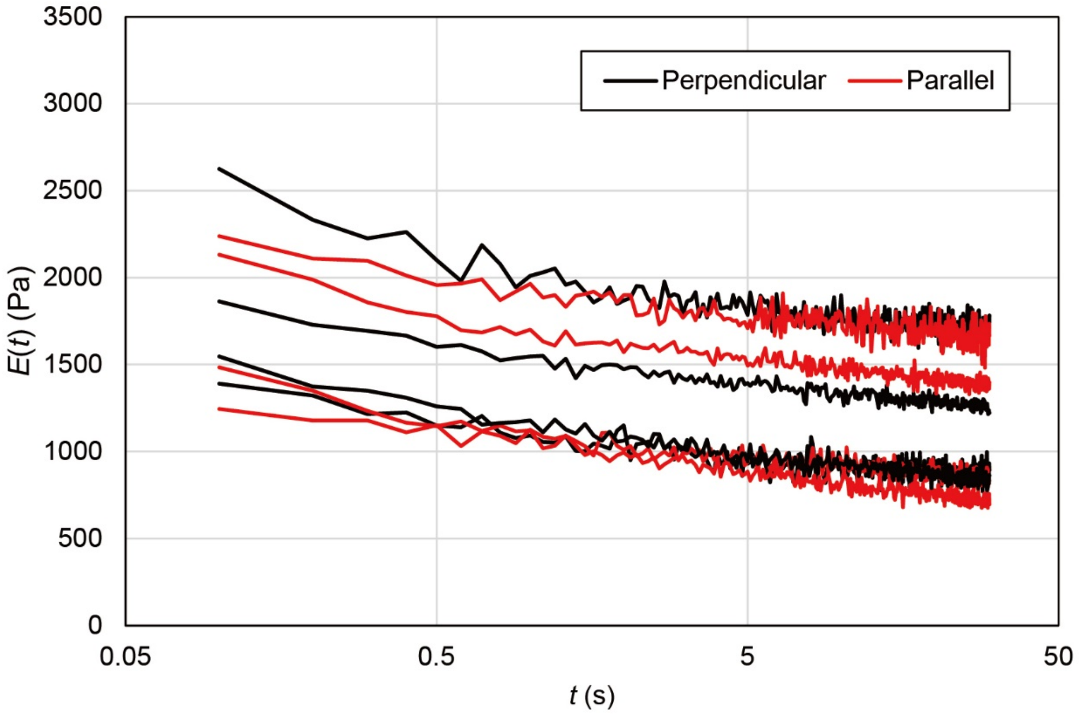

Figure S3. Relaxation moduli of MCCG in the perpendicular and parallel directions. All measured results are summarized in the figure (n =4).

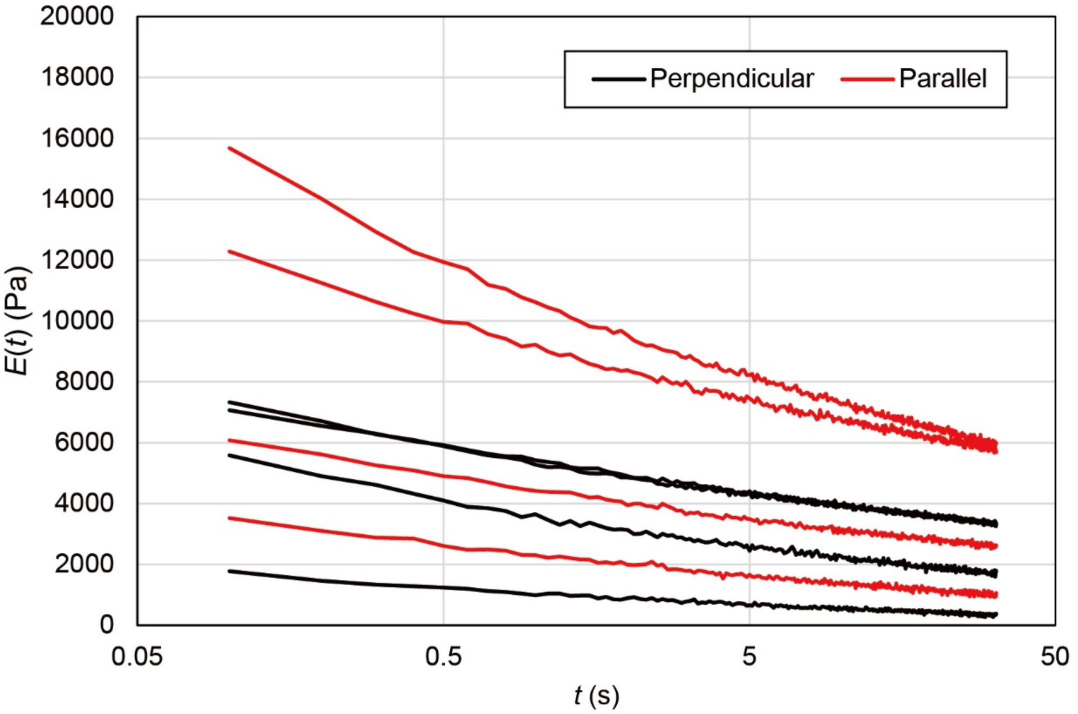

Figure S4. Relaxation moduli of CT in the perpendicular and parallel directions. All measured results are summarized in the figure (*n* =4).

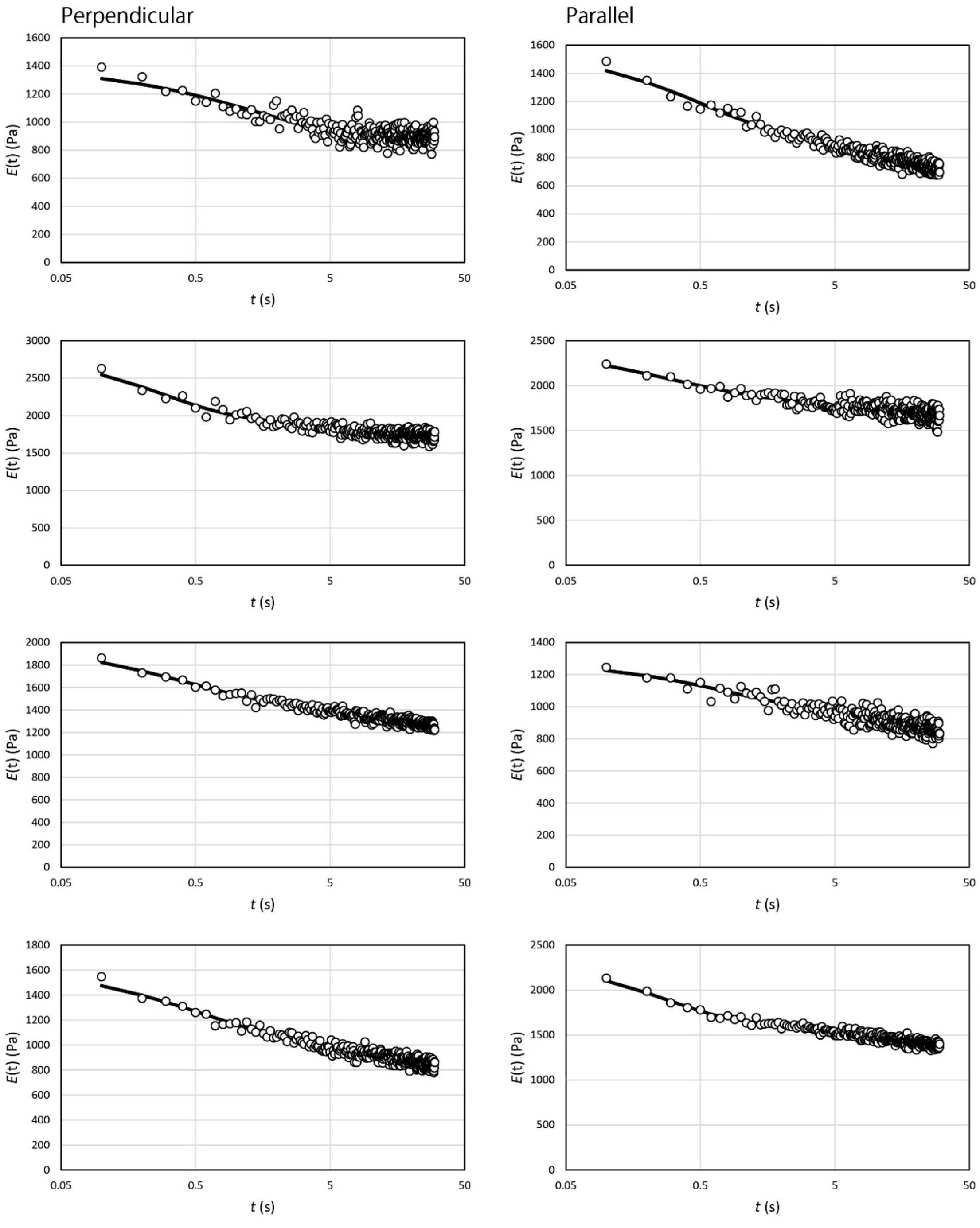

Figure S5. Curve fitting the relaxation moduli of MCCG in the perpendicular and the parallel directions with the equation 3.

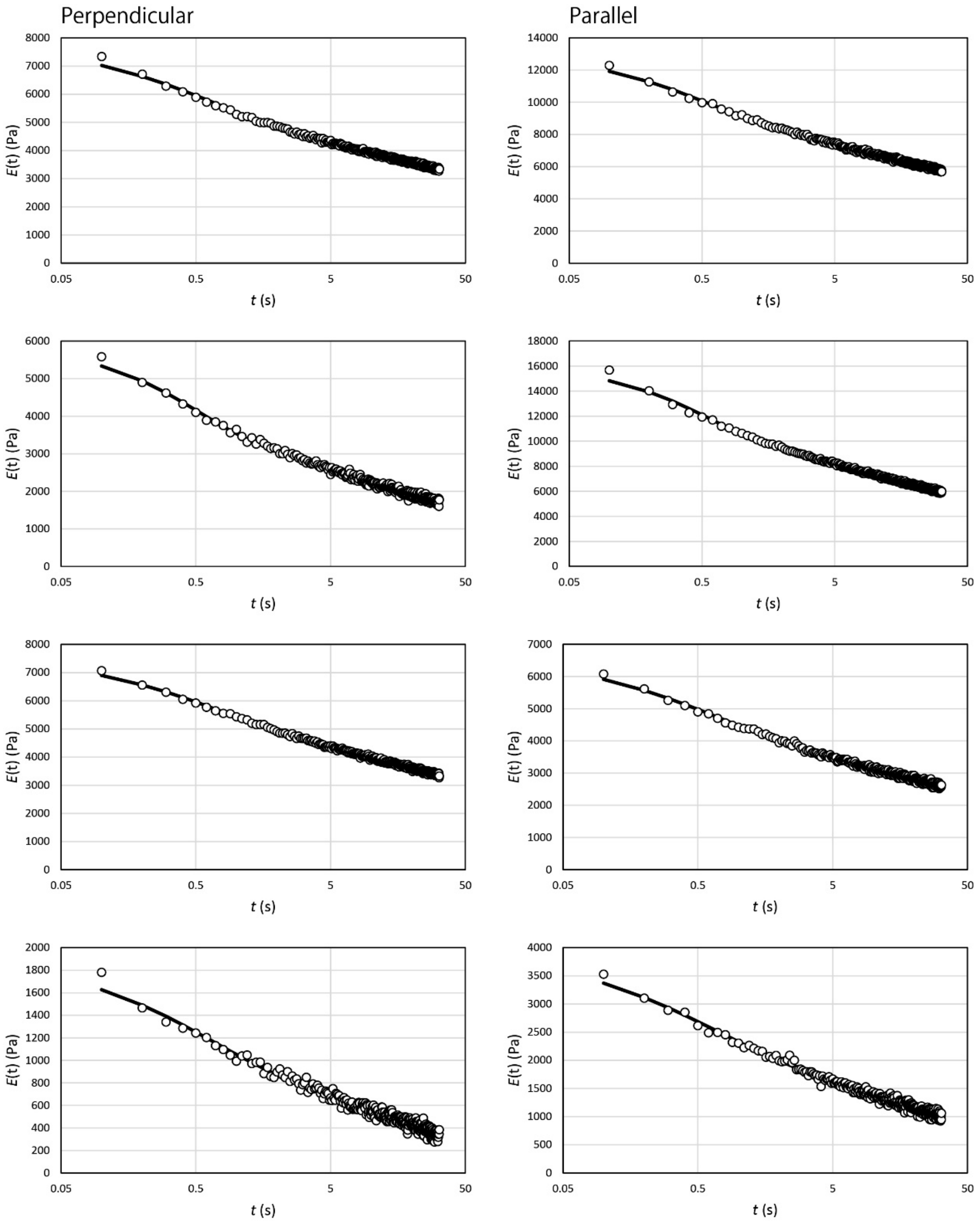

Figure S6. Curve fitting the relaxation moduli of CT in the perpendicular and the parallel directions with the equation 3.

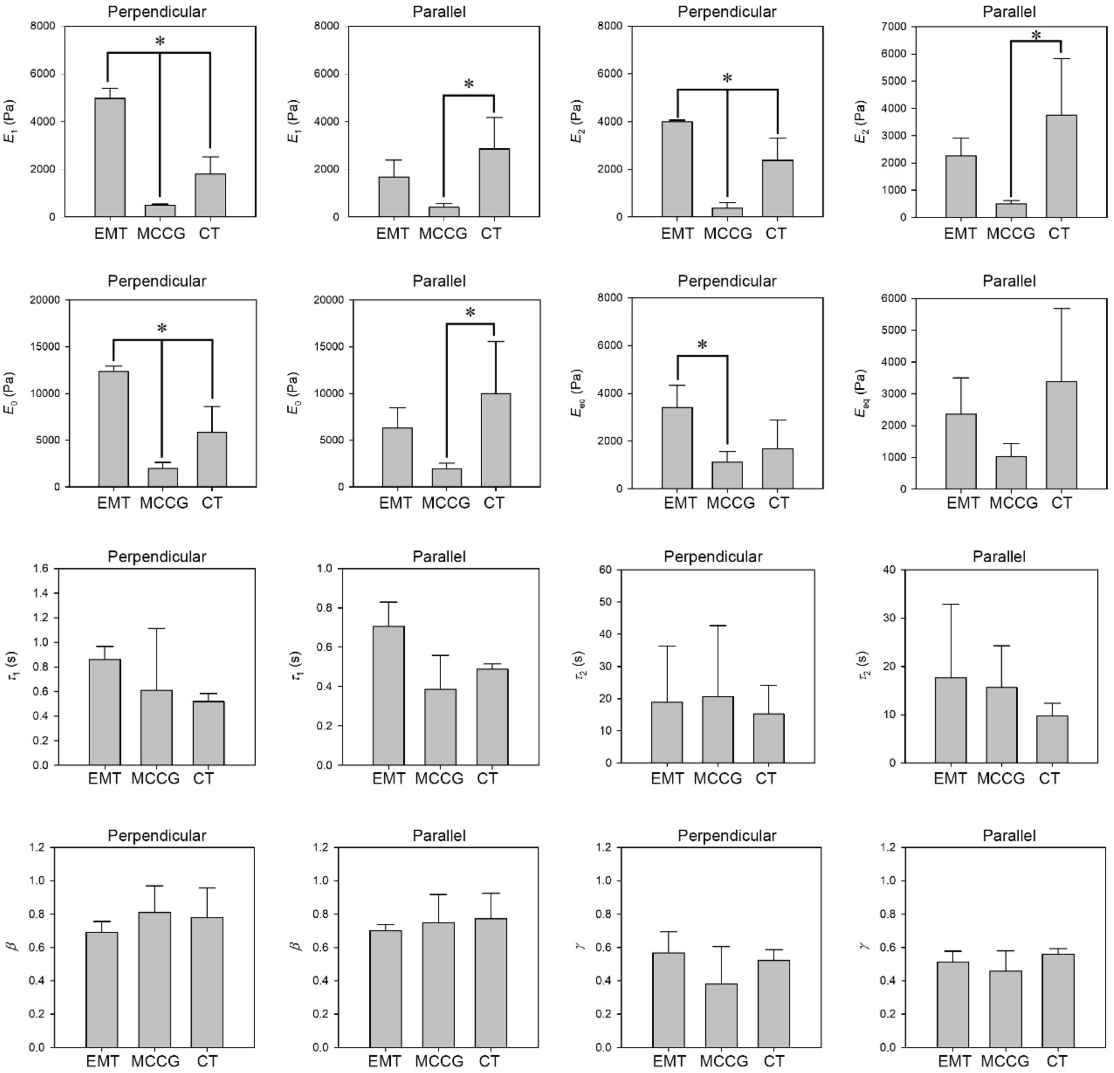

Figure S7. Comparing the relaxation parameters of EMT, MCCG and CT in the perpendicular and the parallel directions. Error bar indicates standard deviation (*n* = 3 for EMT, and *n* = 4 for MCCG and CT). Asterisk indicates significant differences of $p < 0.05$. The comparison performed by using ANOVA test and post-hoc Tukey's test.

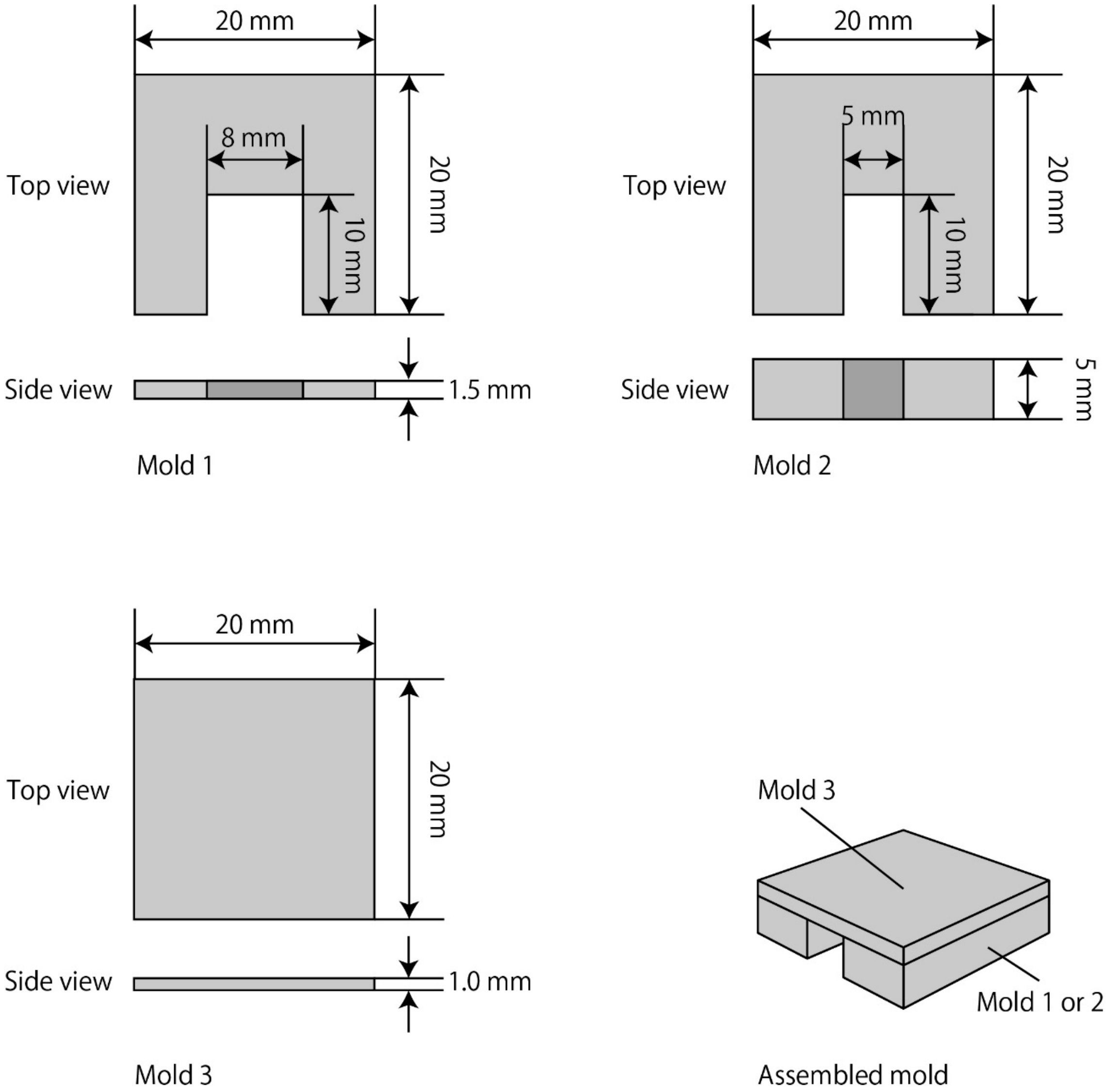

Figure S8. Schematic illustrations of molds for preparing MCCGs.

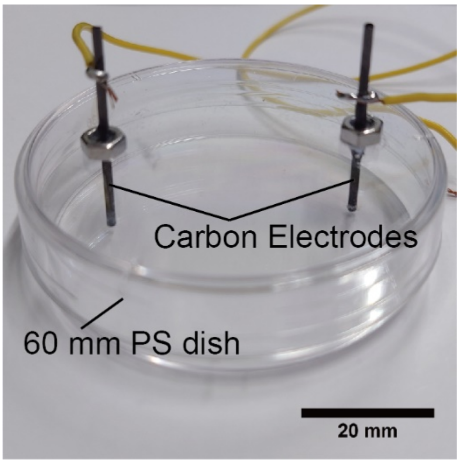

Figure S9. A photograph of the hand-maid electrode for electrostimulation to EMT.
